# Comparison of ligand binding and conformational stability of human calmodulin with its homolog from the malaria parasite *Plasmodium falciparum*

**DOI:** 10.1096/fba.2020-00013

**Published:** 2020-08-09

**Authors:** Tünde Juhász, József Kardos, Zsolt Dürvanger, Veronika Harmat, Károly Liliom

**Affiliations:** ^1^ Institute of Materials and Environmental Chemistry Research Centre for Natural Sciences Budapest Hungary; ^2^ Department of Biochemistry Institute of Biology ELTE Eötvös Loránd University Budapest Hungary; ^3^ Laboratory of Structural Chemistry and Biology Institute of Chemistry Eötvös Loránd University Budapest Hungary; ^4^ MTA‐ELTE Protein Modelling Research Group Budapest Hungary; ^5^ Department of Biophysics and Radiation Biology Faculty of Medicine Semmelweis University Budapest Hungary

**Keywords:** binding affinity, inhibitor development, protein stability, protein structure, target activation

## Abstract

Calmodulin (CaM), the key calcium sensor of eukaryotic cells regulating a great number of target proteins, belongs to the most conserved proteins. We compared function and properties of CaMs from two evolutionarily distant species, the human (*Homo sapiens*) representing vertebrates, and the malaria parasite *Plasmodium falciparum* (Pf). The biophysical characterization revealed higher stability of Pf CaM attributed to the more stable C‐terminal domain in both Ca^2+^ free and saturated states. In vitro binding and functional assays demonstrated that human and Pf CaM exhibit similar biochemical features involving small molecule inhibitor binding and target enzyme activation as illustrated by comparable affinities differing only within a factor of three. It has been reported that CaM antagonists proved to be antimalarials, so Pf CaM could be a potential target to combat malaria parasites. Indeed, we observed that phenotypically active compounds from the Malaria Box could show inhibitory action on Pf CaM, among them the most potent exhibited comparable inhibition to known antagonists of vertebrate CaM. However, based on the minor binding differences in Pf CaM to human CaM, we conclude that CaM is an unsuited target for human intervention against malaria, due to the likely interference with the host protein.

**Abbreviations**
ADP: adenosine diphosphate
ANS: 8‐anilinonaphthalene‐1‐sulfonic acid
ATP: adenosine triphosphate
BSA: bovine serum albumin
CaM: calmodulin
CaN: calcineurin
CD: circular dichroism
C‐domain: C‐terminal domain
dansyl: 5‐(dimethylamino)naphthalene‐1‐sulfonyl
DSC: differential scanning calorimetry
DSF: differential scanning fluorimetry
DTT: dithiothreitol
EGTA: ethylene glycol‐bis(β‐aminoethyl ether)‐*N*,*N*,*N*′,*N*′‐tetraacetic acid
HEPES: 4‐(2‐hydroxyethyl)‐1‐piperazineethanesulfonic acid
ITC: isothermal titration calorimetry
mant‐cGMP: 2′‐(N‐methylanthraniloyl)guanosine 3′,5′‐cyclicmonophosphate
MLCK: myosin light chain kinase
MMV: Medicines for Malaria Venture
N‐domain: N‐terminal domain
PAGE: polyacrylamide gel electrophoresis
PDE: phosphodiesterase
Pf: Plasmodium falciparum
pI: isoelectric point
PNPP: p‐nitrophenyl phosphate
SDS: sodium dodecyl sulfate
TFP: trifluoperazine
T_m_: melting point
W7: N‐(6‐aminohexyl)‐5‐chloro‐1‐naphthalenesulfonamide

## 1 INTRODUCTION

Calmodulin (CaM), the ubiquitous intracellular Ca^2+^ sensor of eukaryotic cell, functions in the control of a wide variety of signaling events regulating the activity of a great number of proteins including enzymes, pumps, and ion channels, in a calcium‐dependent manner.

The small (148 aa), evolutionary highly conserved protein contains two homologous globular domains or lobes connected by a flexible central linker. Both lobes are composed of two helix‐loop‐helix EF‐hand motives, allowing the binding of maximum four Ca^2+^ ions.^1^ Despite the homology of the lobes, the C‐ and N‐terminal lobes show differences in folding and structural aspects, and, partly related to this, in their affinity to Ca^2+^. It is well‐documented that in the Ca^2+^‐free apo form, the C‐terminal domain (C‐domain) is more disordered, and thus has a lower stability than the more folded N‐terminal lobe. Upon Ca^2+^‐binding, the stability of both domains raises dramatically. The C‐terminal domain bears higher affinity Ca^2+^‐binding sites, so it is expected to possess higher stability in Ca^2+^‐saturated CaM (CaCaM) as it was proposed in studies using experimental or computational approaches.^2^, ^3^ In contrast, higher stability for the N‐terminal domain (N‐domain) in both apo and holo CaM forms was suggested based on thermal melting profiles observed with differential scanning calorimetry.^4^, ^5^

Ca^2+^‐binding to CaM results in a global conformational change involving rearrangement of the helices as well as additional helix formation. Moreover, this conformational change leads to exposure of hydrophobic patches,^6^ opening binding pockets for its target proteins.^7^ Typical CaM binding sites are ~25 amino acid long sequences^8^ which can fold into basic amphipathic helices, forming both hydrophobic and ionic interactions with CaM.^9^ Target peptides are bound in the central channel of CaM, wrapped around by the protein. The structural constraints for a CaM‐binding motif are not so strict,^8^ thus target orientation, distances of their anchoring residues and orientation of the CaM lobes can vary within the CaM‐target complexes (reviewed in Ref. [10, 11, 12]). Nevertheless, several proteins can bind to CaM at low Ca^2+^ concentrations or independently of calcium (IQ motifs).^13^ Binding to apoCaM involves the C‐domain adopting a semi‐open conformation, that is, partial opening of its hydrophobic pocket.

There are several synthetic small molecules like the antipsychotic drug TFP or the widely used W5‐W13 series as well as a few natural substances mostly from fungi or plants with known anti‐CaM activity,^14^ most of them occupying the target peptide binding site in Ca^2+^‐loaded CaM.

The fact that CaM ‐antagonists like W7 were able to inhibit malaria parasites was reported ~30 years ago (reviewed in Ref. [15]). More than 200 million people are affected each year by malaria, with around 435 000 related deaths in 2017 (WHO, World Malaria Report, 2018).^16^ The causative agents of the disease are the *Plasmodium* species, the most dangerous form is caused by *Plasmodium falciparum* (Pf). The current treatments, the artemisinin combination therapies show decreased efficiency with detected Pf resistance to artemisinin in Asia,^17^, ^18^ so there is an urgent need for novel antimalarial drugs.^16^, ^19^, ^20^ To help treat neglected diseases like malaria, a collection called Malaria Box was assembled by the Medicines for Malaria Venture (MMV) foundation. The Malaria Box contains 400 phenotypically active compounds with diverse chemical composition, however, with mostly unknown mechanism of action.^21^

According to general considerations in protein structure‐function studies, it is accepted that a fine‐tuned balance between flexibility and stability controls function. In the present study we examined the biochemical and the biophysical properties of two homologs of the highly conserved protein CaM from two distantly related species, *Homo sapiens* and Pf. Our findings on their stability and ligand‐binding ability can contribute to understand how CaM works as a versatile tool regulating the action of many target proteins. The potential of CaM as a drug target in development compounds against malaria is also discussed.

## 2 MATERIALS AND METHODS

### 2.1 Protein preparation

Human recombinant CaM was expressed in *E coli* and purified using phenyl‐sepharose affinity chromatography as described previously.^22^, ^23^ The gene encoding Pf CaM (Uniprot P62203, CALM_PLAF7) was synthesized and subcloned into the vector pET‐3d by Eurofins MWG Operon (Ebersberg, Germany). Pf CaM was expressed in *E coli* as described for the human protein, and was purified using phenyl‐sepharose chromatography with the following minor modifications: the crude *E coli* extract was applied to the column in the presence of 5 mmol/L CaCl_2_ then washed with a buffer containing 1 mmol/L CaCl_2_ followed by 0.1 mmol/L CaCl_2_ then 0.1 mmol/L CaCl_2_ + 1 mol/L NaCl, finally eluted with 1 mmol/L EGTA. During the washing steps, some Pf CaM eluted. Protein purity was checked by SDS‐PAGE, and CaM concentration was determined by measuring the absorbance at 280 nm using an extinction coefficient of 0.178 and 0.177 for the 1 mg/mL solution of human CaM and Pf CaM, respectively, as calculated at https://web.expasy.org/protparam. For Pf CaM, the correct size of the protein product was verified by mass spectrometry. The purified protein was stored after buffer exchange in the *standard assay buffer* (10 mmol/L HEPES, 100 mmol/L KCl, pH 7.2) at −18°C.

### 2.2 Circular dichroism spectroscopy

CD spectra were recorded on Jasco J‐720 and J‐810 spectropolarimeters (Jasco) using quartz cuvettes with pathlengths of 1 mm in the far‐UV and 1 cm in the near‐UV range. Samples were measured in the standard assay buffer complemented with 1 mmol/L EGTA or CaCl_2_ at a protein concentration of 5‐8 μmol/L in the far‐UV and 210 μmol/L in the near‐UV. Spectra were recorded at least three times at a scan speed of 20 nm/min using a bandwidth and response time of 1 nm and 4 seconds, respectively. Secondary structure content was estimated using the BeStSel method available at http://bestsel.elte.hu.^24^ Melting curves were measured in standard assay buffer or 25 mmol/L phosphate, pH 7.0, in the presence of 1 mmol/L EGTA or saturating levels of CaCl_2_ monitoring the ellipticity at 222 nm for secondary structure changes or at 280 nm to monitor the signal of Tyr residues. A heating rate of 1 or 2°C/min was used. In a well‐sealed 1 mm‐cell, heating up to 110°C was possible.

### 2.3 Electrophoresis

Native PAGE was performed in continuous mode (6% acrylamide gels without stacking gel) using a buffer system of pH 7.4 containing imidazole (43 mmol/L) and HEPES (35 mmol/L).^25^ This system is useful for proteins with a net negative charge at physiological pH, such as CaM with a pI value as low as 4.1. Typically, 10 μL samples were loaded. Samples for SDS‐PAGE were heated at 90°C for 5 minutes in the presence of 0.8% SDS. Standard discontinuous SDS‐PAGE according to Laemmli^26^ was performed using 15% acrylamide gels.

### 2.4 Differential scanning calorimetry

Thermograms were measured in the standard assay buffer complemented with 1 mmol/L EGTA or CaCl_2_ using a VP‐DSC microcalorimeter (MicroCal). Protein samples at 0.75 mg/mL were heated at a scan rate of 1°C/min with no feedback. Data analysis was performed with the Origin software provided by MicroCal.

### 2.5 Differential scanning fluorimetry

Denaturation curves were determined by detecting intrinsic protein fluorescence as a function of temperature using the Prometheus instrument (NanoTemper Technologies). Protein samples at 1.0‐1.5 mg/mL were heated at a scan rate of 5°C/min up to 110°C in the standard assay buffer using the designated set and capillaries. Data analysis was performed with the built‐in analysis software.

### 2.6 Fluorescence spectroscopy

Spectra were collected using a Jobin Yvon Fluoromax‐3 spectrofluorometer at 25°C in the standard assay buffer containing either 1 mmol/L EGTA or 1 mmol/L CaCl_2_. Spectra were recorded three times, averaged, and corrected by subtracting the appropriate blank. Tyrosine fluorescence was excited at 274 nm, the emission was detected between 285 and 370 nm, and the intensities were recorded at 303 nm. In ANS binding assays, 10 μmol/L of ANS (8‐anilinonaphtalene‐1‐sulfonic acid, Fluka, 10417) was excited at 388 nm in the presence of 2 μmol/L CaM in standard assay buffer, and the emission was monitored from 410 to 600 nm. In experiments with dansyl‐labeled CaM, the fluorophore was excited at 340 nm, and the emission was monitored from 400 to 600 nm.

### 2.7 Isothermal titration calorimetry

Thermodynamic parameters of the interaction of CaCaM with melittin, W7, and TFP, were examined using a VP‐ITC or ITC_200_ instrument (MicroCal). W7 (A3281) and TFP (T6062) were purchased from Sigma, and melittin was synthesized by EZ Biolab (Carmel, IN, USA). Measurements were performed at 25°C in the standard assay buffer containing 1 mmol/L CaCl_2_, and 1% DMSO when measuring W7, TFP, and the MMV compound. Aliquots of the protein (~200 μmol/L) were injected into the ITC cell containing 20‐25 μmol/L peptide or small molecule in the same buffer. Titration curves were fitted with the models provided by the built‐in Origin software (MicroCal.).

### 2.8 Calcineurin activity assay

Calcineurin (PPase‐2B, CaN) was purchased from Promega (V6361, isolated from bovine brain). Dephosphorylation of the substrate p‐nitrophenyl phosphate (PNPP, Sigma, 1040) was followed by monitoring the increase in absorbance at 405 nm using a Perkin Elmer EnSpire microplate reader. The CaN activity was assayed by measuring the initial velocities at 28°C in 50 mmol/L Tris, pH 7.5 containing 0.001 unit/μL CaN, 0.1 mg/mL BSA, 20 mmol/L PNPP, 1 mmol/L NiCl_2_, and various amounts of CaM to determine dose‐response curves for CaN activation by CaM. For testing inhibition of CaM function, 10 nmol/L CaM and various amounts of the small molecules were used.

### 2.9 PDE activity assay

Phosphodiesterase I, 3′,5′‐cyclic‐nucleotide‐specific (PDE) was purchased from Sigma (P9529, isolated from bovine brain) and mant‐cGMP from Calbiochem (370668). PDE activity was measured fluorometrically following the consumption of mant‐cGMP as described by Johnson et al,^27^ so as mant‐cGMP was excited at 280 nm and emission was monitored at 450 nm. The reaction was followed at 25°C in standard assay buffer containing 5 mmol/L MgCl_2,_ and either 1 mmol/L CaCl_2_ or 1 mmol/L EGTA (as a control). Mant‐cGMP, PDE, and CaM concentrations were 10 μmol/L, 6.5 nmol/L, and 150 nmol/L, respectively. For Pf CaM, PDE activity was also tested upon addition of TFP (1 μmol/L) or melittin (200 nmol/L). The initial velocities of substrate hydrolysis were measured in the absence and in the presence of CaM yielding the basal and the CaM‐dependent activities, respectively.

### 2.10 MLCK activity assay

Myosin light chain kinase (MLCK) was purchased from Sigma (M9197, human, recombinant, GST‐tagged). Enzyme activity was measured in standard buffer complemented with 0.5 mmol/L CaCl_2_, 2.5 mmol/L MgCl_2,_ and 0.1 mg/mL BSA. When measuring activation by CaM, 20 μL reaction mixtures contained 3.5 ng/μL MLCK, 0.1 mg/mL MLCK substrate (Sigma SCP0196), 50 μmol/L ATP, 0.5 mmol/L DTT, and various amounts of CaM. Samples were incubated for 30 minutes at room temperature then ADP formed during the kinase reaction was detected using the ADP‐Glow Kinase Assay kit (Promega) according to the manufacturer's instructions. Luminescence values proportional to ADP concentration were normalized to maximal intensities obtained at saturating CaM concentrations.

### 2.11 Data analysis and statistics

Statistical analysis and nonlinear regression curve fitting was done by Origin software (OriginLab). Data are presented as mean ± SD, and *n* indicates the number of samples tested.

## 3 RESULTS

### 3.1 Structure and Ca^2+^‐binding properties of CaM from *P falciparum*

To characterize its properties, we recombinantly expressed, and purified Pf CaM for the first time. The standard purification method based on phenyl‐sepharose chromatography exploiting the hydrophobic character of the Ca^2+^‐loaded protein could be used for Pf CaM, too.

#### 3.1.1 Primary structure

**FIGURE 1:**
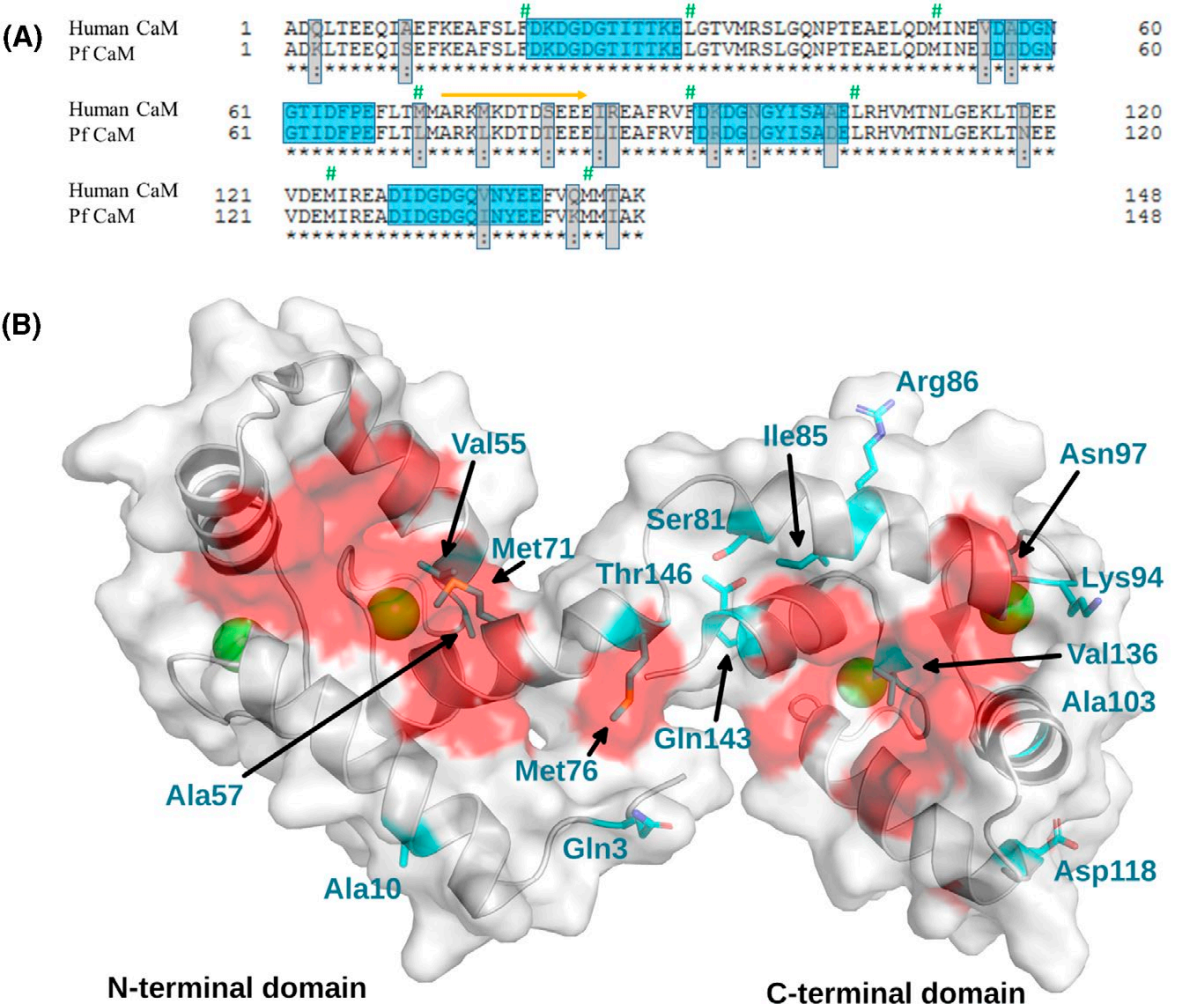
Sequence comparison of human and Pf CaM A, Sequence alignment of human and Pf CaM. Nonidentical residues and Ca^2+^‐binding loops are highlighted by gray and blue boxes, respectively. Similar substitutions are marked by colon. Residues forming the hydrophobic pockets are labeled by green hashes, and the flexible central linker by an orange arrow. Alignment was performed using the uniprot website (https://www.uniprot.org). B, Location of the nonidentical residues on the structure of CaM. The structure of human CaM is shown (pdb code:  2MGU). Differences between human and Pf CaM are highlighted by cyan and shown with sticks, and Ca^2+^ ions are shown as green balls. Hydrophobic surfaces participating in ligand binding are shown in red.

CaM is highly conserved which is clearly illustrated by the fact that CaM from all mammalian, even from all vertebrate species, are identical. The sequence identity between mammalian and the evolutionary distant Pf CaM is 89% (Figure 1). Moreover, 13 of the 16 nonidentical amino acids are similar substitutions. The three nonsimilar positions, Arg86, Ala103, and Thr146 according to the human sequence, all reside in the C‐terminal domain containing the high affinity Ca^2+^‐binding sites, EF‐hands III and IV. Among these, residue103, Ala in human, and Asp in Pf CaM, respectively, is part of the Ca^2+^‐binding loop of EF‐hand III, however, its side chain is not involved in Ca^2+^ binding in view of the canonical consensus sequence of the motif.

#### 3.1.2 Secondary structure

**FIGURE 2:**
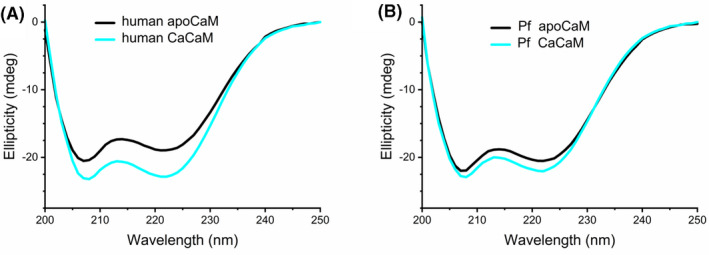
Secondary structure comparison. Far‐UV CD spectra of human (A) and Pf (B) CaM in the presence of added 1 mmol/L EGTA (apoCaM) or 1 mmol/L CaCl_2_.(CaCaM). Spectra were recorded at a protein concentration of 8 µmol/L

The huge sequence similarity between human and Pf CaMs suggests similar structural properties of the two proteins. Indeed, far‐UV CD spectra indicated an all‐ alpha helical structure for both (Figure 2). Mammalian CaM is known to undergo conformational changes upon Ca^2+^ binding, which is accompanied by 21% elevation of the CD signal.^28^ In agreement with this result, we observed 20% increase in ellipticity, and estimated an elevation of the α‐helix content from 42% to 50% for the human protein upon Ca^2+^ saturation. In contrast, smaller spectral differences were observed for Pf CaM yielding only 8% ellipticity gain, and a change from 44% to 47% in the α‐helix content in the presence of 1 mmol/L Ca^2+^ vs 1 mmol/L EGTA. This finding suggested slight differences in both the basic conformation of the apo‐ and CaCaM and the nature of the conformation change upon Ca^2+^ binding for the two proteins.

#### 3.1.3 Tertiary structure––Intrinsic fluorescence

**FIGURE 3:**
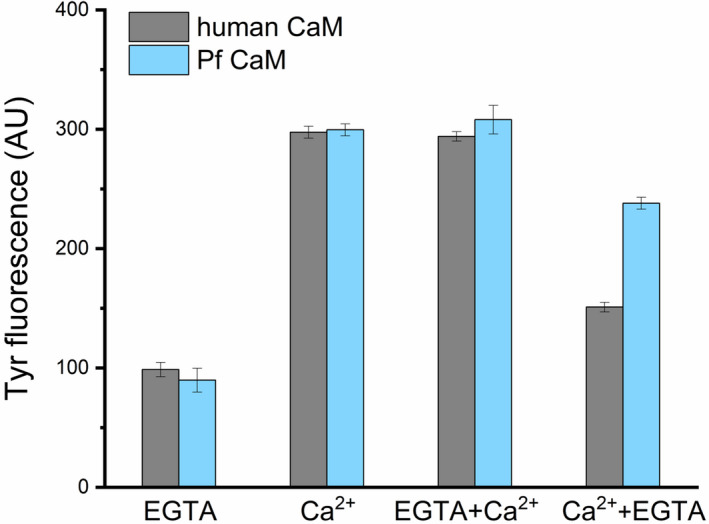
Tyrosine fluorescence of human and Pf CaM. Spectra were recorded at 2 µmol/L protein concentration. Maximal fluorescence intensities at 303 nm were determined in the presence of added 1 mmol/L EGTA (EGTA) or 1 mmol/L CaCl_2_ (Ca^2+^), and added 2 mmol/L CaCl_2_ after incubation with 1 mmol/L EGTA (EGTA + Ca^2+^) or 2 mmol/L EGTA after incubation with 1 mmol/L CaCl_2_ (Ca^2+^ +EGTA)

CaM contains no tryptophan but two tyrosines in the C‐terminal domain, thus exploring the intrinsic fluorescence under various conditions or in the presence of additives can give information about conformational changes involving the Tyr residues in the C‐domain. Indeed, Tyr fluorescence is known to be sensitive to Ca^2+^‐load. Upon saturation of the Ca^2+^‐binding sites III and IV, the fluorescence intensity of CaM was reported to increase by a factor of 2‐3 for mammalian CaMs.^29^ We have measured an approximately threefold elevation in the fluorescence intensity followed at the emission maximum at 303 nm upon excitation at 276 nm for both the human and Pf CaMs in the presence of 1 mmol/L CaCl_2_ compared to 1 mmol/L EGTA (Figure 3). In the presence of excess Ca^2+^, the same spectra were recorded independently whether EGTA was added before. In contrast, adding excess amount of EGTA after incubation with CaCl_2_ resulted in intensities higher than those with added EGTA only (Figure 3). Moreover, this difference was not the same for the two CaMs indicating different Ca^2+^ affinities for the C‐domain in the two proteins.

#### 3.1.4 Tertiary structure––Electrophoretic mobility

**FIGURE 4:**
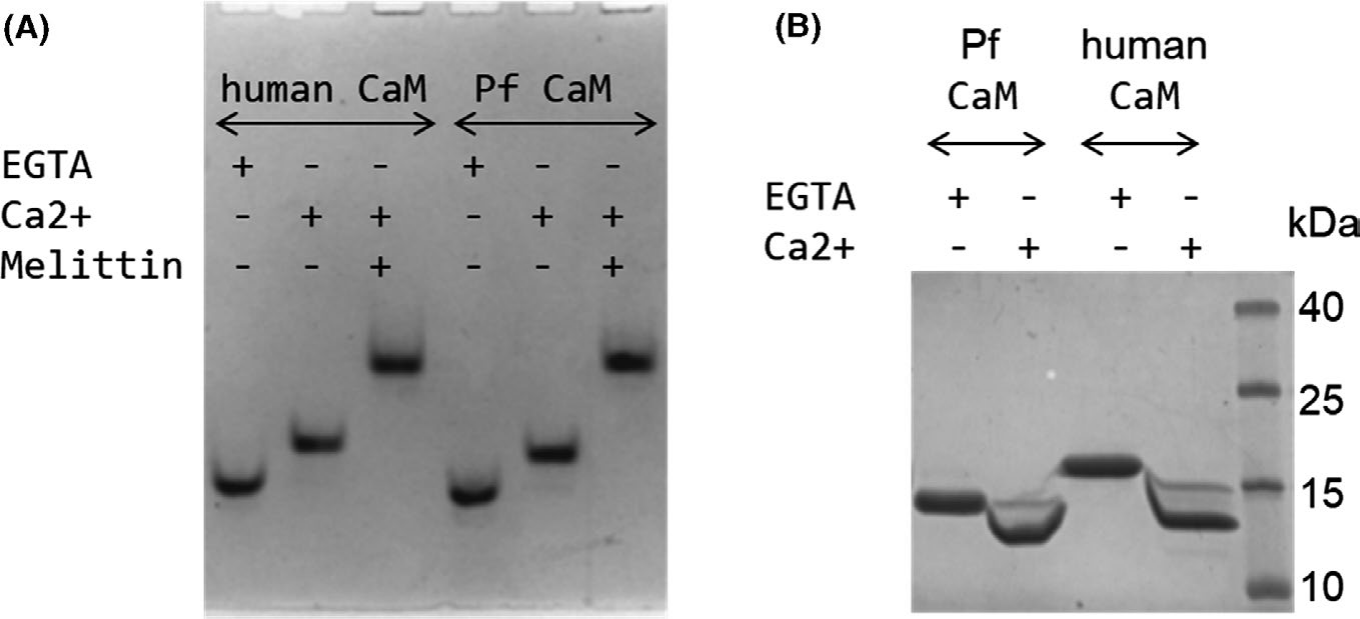
Electrophoretic mobility of human and Pf CaM. Samples of human and Pf CaM were run in the presence and absence of Ca^2+^ using (A) native (Imidazole/HEPES) PAGE, or (B) SDS‐PAGE. A, Migration pattern of 10 µmol/L (1.7 µg) CaM in the presence of 1 mmol/L EGTA or CaCl_2_, and 20 µmol/L melittin. B, Migration pattern of CaM after incubation with 5 mmol/L EGTA or CaCl_2_ using SDS‐PAGE (15% acrylamide gel)

CaM is known for the different mobility of the apo and Ca^2+^‐loaded forms using both native and SDS‐PAGE.^30^, ^31^ Higher mobility of apoCaM over CaCaM in native PAGE can be attributed to the bound positively charged Ca^2+^ ions to the fairly negatively charged apoprotein. The two CaMs showed very similar migration patterns (Figure 4A) in agreement with their identical net charges although Pf CaM possesses one more negatively and positively charged residue each (39 vs 38 Asp + Glu, and 15 vs 14 Arg + Lys out of the total 148 residues for Pf and human CaM, respectively).

Using SDS‐PAGE, the Ca^2+^‐saturated forms migrated faster than the apo forms for both CaMs, but both Pf forms showed higher mobility than the corresponding human CaM forms (Figure 4B). Human and Pf CaM has a molecular weight (*Mw*) of 16.7 and 16.8 kDa, respectively, but only human apoCaM migrated according to its *Mw* while Pf apoCaM exhibited a smaller apparent *Mw* of ~14 kDa. It should be noted that this is not due the improper size of the recombinant Pf CaM as its *Mw* was verified by mass spectrometry resulting a *Mw* of 16798.8 Da identical with the theoretical value (16799.5 Da) within the error of determination (±2 Da). Instead, the unusual mobility might be attributed to differences in stability of the four states of the two proteins resisting to different extent to the denaturing conditions applied upon SDS‐PAGE. Indeed, remarkable stability differences were detected as described in detail in the next section.

### 3.2 Comparison of the stability of human and Pf CaM

Stability of human and Pf CaM was investigated comparing their thermal stability using various methods. Among these, properties of the whole protein can be explored with differential scanning calorimetry and far‐UV circular dichroism while the C‐terminal domain alone can be studied utilizing differential scanning fluorimetry or circular dichroism in the near‐UV region.

#### 3.2.1 Differential scanning calorimetry

**FIGURE 5:**
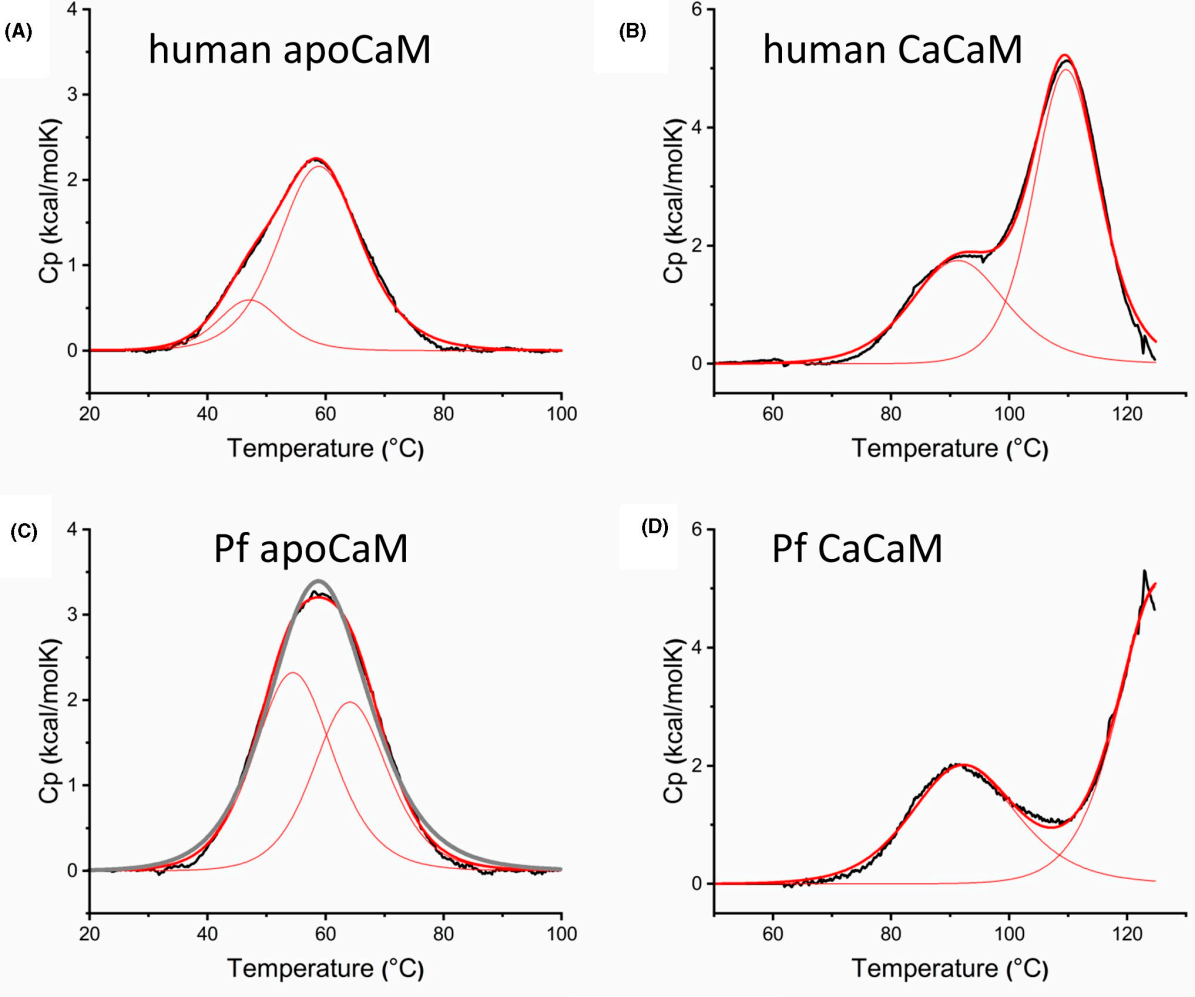
Heat stability of human and Pf CaM. Thermal stability was studied by DSC in standard assay buffer. Excess transition heat capacity *C*p is plotted against temperature (black line), fitted with a two‐state model, and deconvoluted to two individual transitions. The fitted and deconvoluted curves are shown as thick and thin red lines, respectively. For comparison, a fit assuming one single transition is also shown for Pf apoCaM (C, gray line). The calculated thermodynamic parameters are listed in Table 1

DSC is a useful technique to study protein stability and also to determine related thermodynamic parameters. It is known that for the mammalian protein, DSC melting curves display two transitions for both apo and CaCaM corresponding to the two domains with different stability. The difference is more pronounced for the Ca^2+^‐loaded form where the two peaks are clearly separated while apoCaM exhibits broad, overlapping peaks.^2^, ^4^ Our results with human CaM agree well with these findings (Figure 5A,B). In general, the behavior of Pf CaM shared the above characteristics (Figure 5C,D), however, several differences were also revealed when compared to the human protein. For Pf CaCaM, the two transitions are almost fully separated (Figure 5D) due to the increased *T*_m_ of the second transition. In contrast, the two transitions seemed to merge to one for Pf apoCaM (Figure 5C) so that assuming a single transition resulted in an acceptable fit. Nevertheless, fitting the data to two transitions yielded a better fit (Figure 5C) suggesting that the two domains melt separately in Pf apoCaM as well. For all peaks except the one for human apoCaM first transition, the calculated calorimetric and van't Hoff enthalpies were in good agreement (Δ*H* vs Δ*H*_V_ in Table 1) indicating a simple two‐state unfolding process for these transitions. As for the exception, the first transition assigned to the C‐domain in mammalian apoCaM displayed a low‐*T*_m_ and low‐intensity peak (Figure 5A) suggesting a less folded protein part. In contrast, Pf apoCaM seems to be more folded as demonstrated by higher *T*_m_ and Δ*H* values comparable to the values of the apo‐N‐domain (Table 1). These results suggested that alterations in the C‐domain provided higher stability for Pf apoCaM. Furthermore, ~15 degrees higher *T*_m_ values were observed for the second transition of Pf CaCaM over human CaCaM while similar *T*_m_ values were detected for the first transition.

**TABLE 1:** Thermodynamic parameters for the thermal denaturation of human and Pf CaM determined by DSC, CD, and DSF. Note that heating rate may differ (as it is 1°C/min for DSC and CD while 5°C/min for DSF

Method	Parameter	Human apoCaM	Human CaCaM	Pf apoCaM	Pf CaCaM
DSC	*T*_m1_ (°C)	46.7 ± 0.1	91.6 ± 0.1	54.7 ± 0.2	92.6 ± 0.1
	Δ*H_1_* (kcal/mol)	6.5 ± 0.4	37.7 ± 0.6	41.2 ± 1.2	47.8 ± 0.3
	Δ*H_v1_* (kcal/mol)	57.2 ± 1.1	49.0 ± 0.7	48.1 ± 0.4	44.5 ± 0.3
	*T*_m2_ (°C)	59.2 ± 0.1	109.8 ± 0.1	64.3 ± 0.1	125.9 ± 0.2
	Δ*H_2_* (kcal/mol)	44.4 ± 0.3	73.9 ± 0.5	33.4 ± 1.2	97.9 ± 2.2
	Δ*H_v2_* (kcal/mol)	44.5 ± 0.2	78.5 ± 0.5	53.5 ± 0.6	65.7 ± 0.8
CD	*T*_m_ (°C)	50.8 ± 0.1		54.4 ± 0.2	
	*T*_m_ (°C)^a^	47.1 ± 0.1		51.2 ± 0.1	
DSF	*T*_m_ (°C)	38.9 ± 0.5		51.2 ± 0.6	

^a^ In phosphate buffer of low ionic strength.

Related to the melting order of domains in the Ca^2+^‐loaded protein, the available data in the literature are controversial. Several studies proposed higher stability for the N‐domain in both apo‐ and CaCaM attributing the transitions with higher heat capacities to the N‐lobe.^4^, ^5^, ^32^ In contrast, other authors suggested a reverse order of stability in apo‐ and CaCaM ^2^, ^3^ arguing that higher Ca^2+^‐affinity provides higher stability for the C‐domain in CaCaM. Monitoring heat stability exploiting Tyr residues located in the C‐domain only could provide evidence in this question. Unfortunately, normal benchtop instrumentation rarely allows measuring fluorescence at >90°C‐95°C, temperatures needed to follow full denaturation of CaCaM.

#### 3.2.2 Circular dichroism

Heat‐induced changes in the protein structure were investigated using CD spectroscopy as well, focusing on the apoproteins. At 25°C, CaM is an all‐alpha protein containing α‐helices up to ~50% (Figure 2). DSC and DSF experiments (see above and below) suggested a more ordered C‐domain for Pf apoCaM compared to human apoCaM, in line with the estimated higher helix content for Pf apoCaM. Heating up the apoproteins, CD spectra turned from characteristic α‐helical to disordered, indicating complete loss of secondary structure upon denaturation (data are not shown but are in good agreement with spectra reported in Ref. [33]). When cooling the samples back to room temperature, >90% of the helical content could be recovered indicating almost complete renaturation, in agreement with an earlier report on mammalian CaM.^2^ This high but not complete reversibility of the thermal unfolding process was also observed by DSC. Reheating apoCaM, similar thermograms with reduced signals and somewhat down‐shifted *T*_m_ values could be measured (not shown).

**FIGURE 6:**
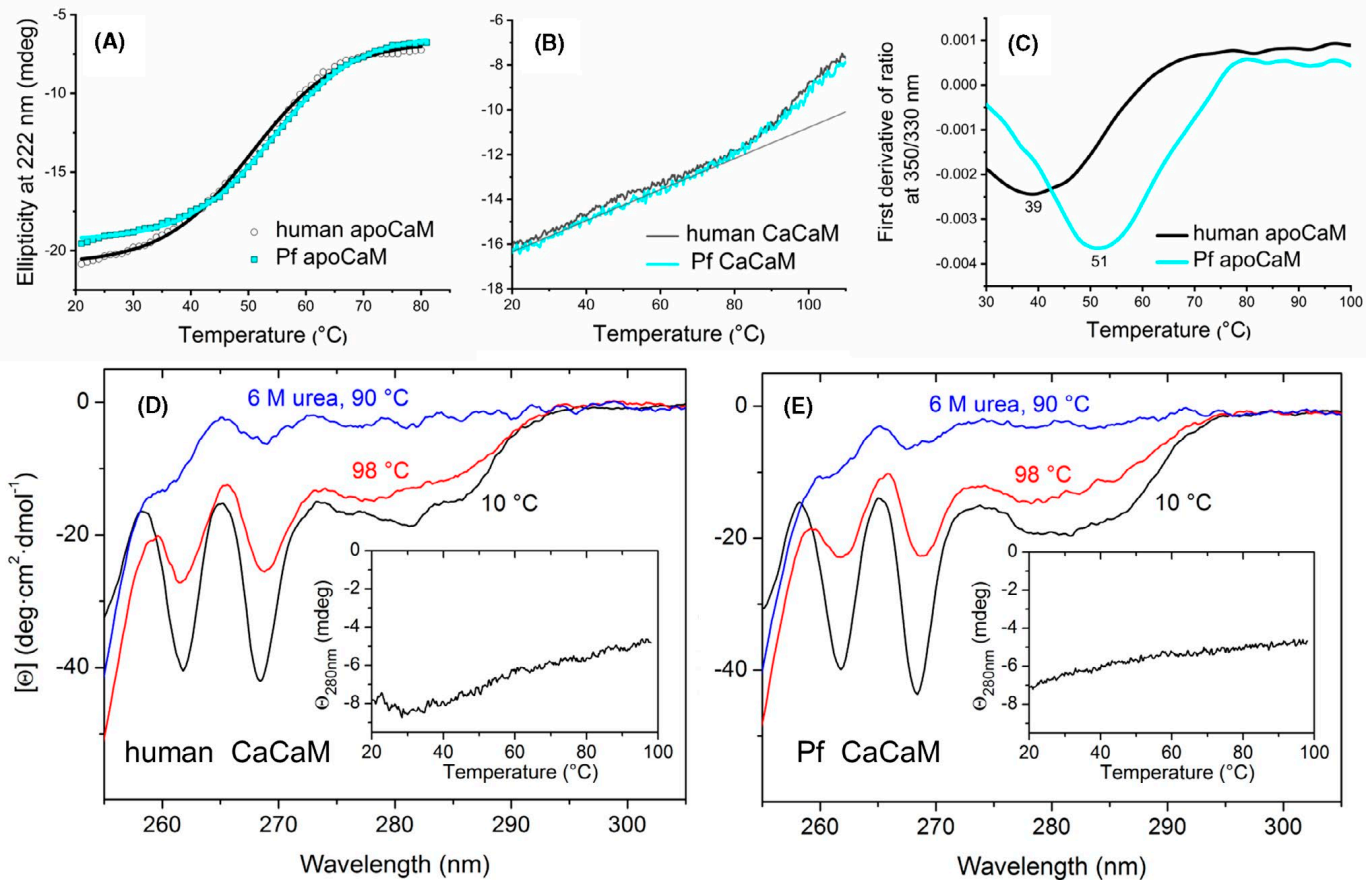
Heat stability of human and Pf CaM studied with CD spectroscopy and differential scanning fluorimetry. A‐B, Ellipticity representing helicity was recorded at 222 nm. For the apoproteins (A), ellipticity is plotted against temperature and fitted to a single sigmoid function for human CaM, (black line) and Pf CaM, (cyan line). For CaCaMs (B), the straight line shows a linear fit to data points in the low temperature range. C, Intrinsic fluorescence was monitored at 330 and 350 nm, the first derivative of the ratio is plotted, and the minimum is determined. D‐E, Near‐UV CD spectra of human and PF CaM under the indicated conditions. Insets show Tyr signal changes recorded at 280 nm upon heating. The parameters obtained (from A and C) can be found in Table 1

Although the CD melting curves were properly fitted to a single sigmoid function (Figure 6A), the unusually wide transition temperature range suggested two overlapping processes as detected by DSC. Nevertheless, 4°C‐5°C higher *T*_m_ values were estimated for Pf apoCaM compared to human apoCaM (Table 1), presumably linked to the more stable C‐domain in Pf apoCaM as seen by other methods.

Thermal scans following helicity at 222 nm were also recorded for the Ca^2+^‐loaded forms upon heating up to 110°C (Figure 6B). These data, in accordance with DSC curves (Figure 5B,D) showed a transition leading to significant helicity loss that is unfolding of a protein part starting at ~80°C, indicated by the upward curvature deviating from the linear regime characteristic at lower temperatures, as observed previously.^34^ The linear region with a gentle slope is in line with a continuous loosening of the helical structure without a definite unfolding. In contrast, following Tyr signal of the C‐domain Tyr residues at 280 nm in the near‐UV region up to 98°C, an almost linear and flat temperature dependence was registered (insets in Figure 6D,E) with no sign of an upward deviation above 80°C, which suggested that the C‐domain did not melt up to 98°C. Similarly, perfect linear dependence up to 94°C was observed for the separated C‐domains of the human and Pf proteins when following ellipticity at 222 nm.^35^ Analyzing individual spectra recorded at 10°C and 98°C (Figure 6D,E) it is clear that the loss of the Tyr signal is less remarkable compared to that of the Phe signals at 262 and 268 nm. As five and three out of the eight Phe residues at conserved positions are located in the N‐terminal and the C‐terminal lobes, respectively, the ~50% intensity reduction at 262 and 268 nm from 10°C to 98°C is suggestive of the unfolding of one domain in this temperature range. This is further supported on comparison with the signals in spectra recorded at 90°C in the presence of 6M urea (Figure 6D,E), representing mostly random aromatic side chain environment in a fully denatured state. It should be noted the similar behavior of the human and parasite proteins. All these findings suggest that while the unfolding of CaCaM takes place from ~80°C, the C‐domain containing the Tyr residues preserves its folded state up to 98°C, and thus the second transition developing above 100°C in the DSC thermograms could reflect the melting of the C‐domain.

#### 3.2.3 Differential scanning fluorimetry

CaM stability was also investigated using nanoDSF exploiting further Tyr residues of the C‐domain, here their intrinsic fluorescence. Denaturation curves were determined for the apoproteins (Figure 6C). The melting points extracted from the DSF curves agree well with the *T*_m1_ values obtained from DSC (Table 1) although the slight variations detected can be attributed to the higher heating rate applied for the DSF method, and to the fact that the DSF device applied here is optimal for tryptophan fluorescence. Altogether, the results confirm again that the transition with lower *T*_m_ value represents melting of the C‐domain in apoCaM. In addition, we can conclude again that the C‐domain of Pf CaM with a *T*_m_ of ~51°C is more stable than that of human CaM showing a *T*_m_ of ~39°C. To probe Ca^2+^‐saturated CaM, samples were heated up to 110°C, the temperature limit of the instrument, however, we were not able to observe a definite transition which could unambiguously be assigned to one of the transitions determined with DSC.

Finally, we should make some remarks about the optimal assay conditions used for testing thermal stability. Our experiments were routinely performed in a standard assay buffer resembling the intracellular milieu (with pH of 7.2, and ion composition of 100 mmol/L KCl), complemented with Ca^2+^ to mimic elevated Ca^2+^‐levels, or EGTA to deplete Ca^2+^. In view of heating up experiments, a HEPES‐based buffer system used here is apparently not the best choice because the high temperature dependence of its p*K*_a_ leads to remarkable shifts in pH at high temperatures. However, the phosphate buffer, commonly used instead, is not compatible with higher Ca^2+^ levels due to precipitate formation. The alternative substituent of phosphate with comparable favorable thermal ionization properties is cacodylate which tolerates Ca^2+^ ions well. However, CaM stability was shown to be sensitive to buffer composition involving pH or ionic strength,^34^ and cacodylate was reported to influence CaM conformation and stability.^9^, ^32^ Thus, using HEPES as buffering agent was reasonable. Nevertheless, some experiments with apoCaM were performed in both HEPES containing 100 mmol/L KCl and 10 mmol/L phosphate buffer with no added salt for purposes of comparison. Following changes in protein secondary structure monitored by far‐UV CD, we obtained similar denaturation curves under the two conditions. Melting temperatures being ~5°C higher in high‐salt over low‐salt buffer (Table 1) are in good agreement with a previous study reporting stabilization of CaM at higher ionic strength.^2^

### 3.3 Ligand binding of human and Pf CaM

Interaction of mammalian CaMs with small molecule antagonists like TFP and W7, and the model target peptide melittin are well‐documented. Similarly, induction of the activity by CaCaM for target enzymes like CaN, PDE, and MLCK is also well‐known, thus these enzymes are widely used to study CaM function in vitro. We investigated whether the binding characteristics of all these interactions alter with Pf CaM.

#### 3.3.1 Small molecule inhibitor binding

**FIGURE 7:**
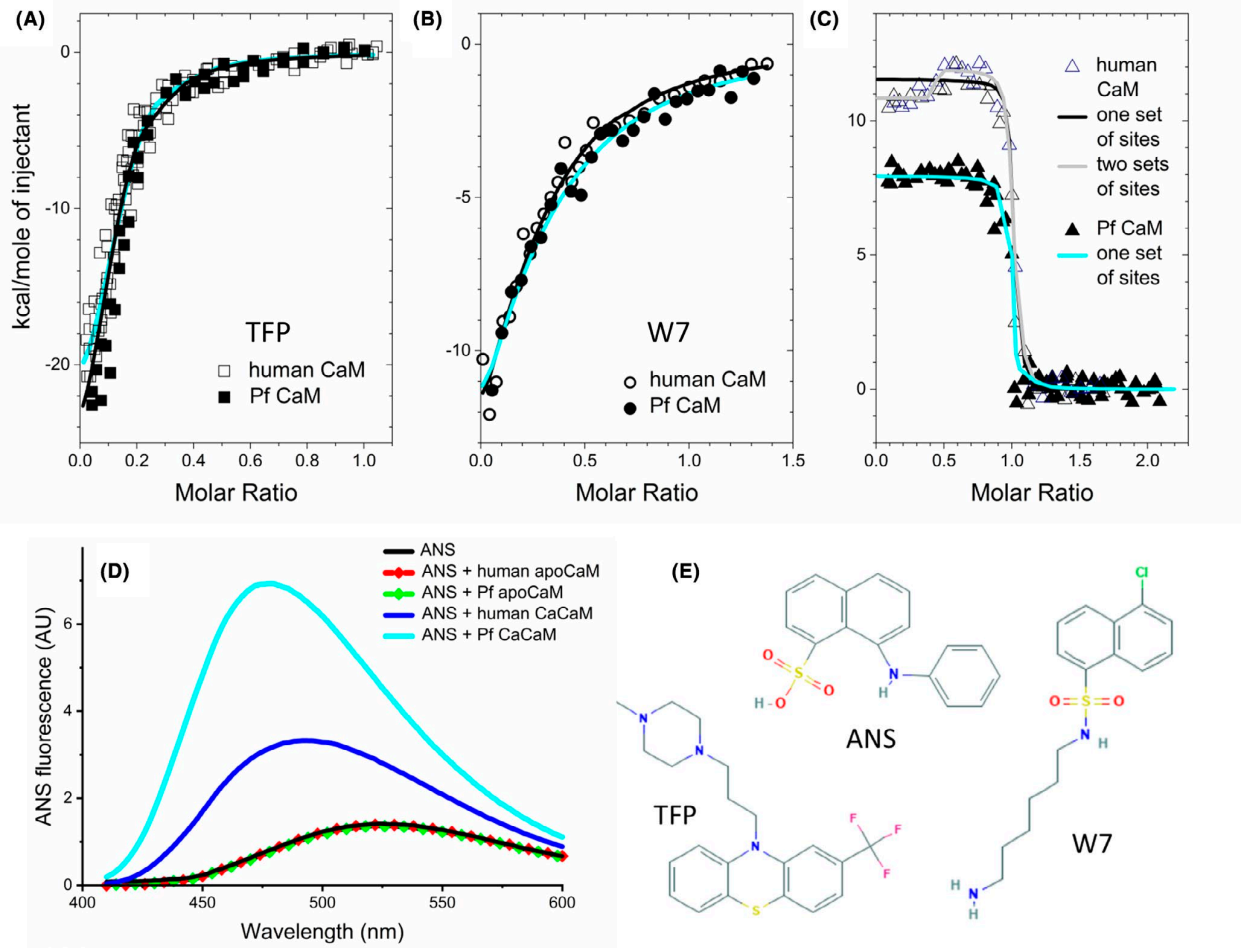
Ligand binding of human and Pf CaM. A‐C, Binding of TFP, W7, and melittin to human and Pf CaM was studied using ITC. Dark and cyan lines show fitted curves for human and Pf CaM, respectively, using the *one set of sites* model. For the human CaM‐melittin binding (C), a fit with the *two sets of sites* model is also shown (gray line). Points are individual data from 2‐4 independent measurements. Parameters obtained from the fit with the *one set of sites* model are A, hCaM‐TFP: n = 7.1 ± 0.2, *K*_d_ = 0.64 ± 0.19 μmol/L, PfCaM‐TFP: n = 7.3 ± 0.2, and *K*_d_ = 1.0 ± 0.23 μmol/L, B, hCaM‐W7: n = 4 fixed, *K*_d_ = 6.7 ± 0.7 μmol/L; PfCaM‐W7: n = 4 fixed, *K*_d_ = 9.4 ± 0.8 μmol/L; C, hCaM‐melittin: n = 1.0 ± 0.1, *K*_d_ < 30 nmol/L, Δ*H* = 11.5 ± 1.0 kcal/mol; PfCaM‐melittin: n = 1.0 ± 0.1, *K*_d_ < 30 nmol/L, Δ*H* = 8.9 ± 0.6 kcal/mol. D, ANS binding to human and Pf CaM. Spectra represent ANS (10 μmol/L) fluorescence in the presence and absence of CaM (2 µmol/L). Note that spectra of both apoproteins totally overlap with the spectrum of free ANS, and ANS fluorescence is not affected by the Ca^2+^ level. E, Chemical structures of the small molecule compounds used in the binding assays. Images were taken from PubChem (https://pubchem.ncbi.nlm.nih.gov)

For TFP, an early study indicated two high‐affinity and numerous low‐affinity, Ca^2+^‐independent sites.^36^ Crystal structures of the CaCaM‐TFP complex showed 1, 2, or 4 small molecules bound both to the hydrophobic clefts as well as to interdomain regions of the protein.^37^, ^38^, ^39^ TFP was reported to bind to mammalian CaM with an affinity of ~1‐5 μmol/L, and a stoichiometry of up to 5 in solution.^40^ Titrating TFP with CaM using ITC herein, calorimetric curves indicated binding of several TFP molecules to each CaM with similar affinity (Figure 7A). Fitting with *one set of sites* model provided *K*_d_ values of ~1 μmol/L with a stoichiometry of 6‐8 for both human and Pf CaMs. The rather high stoichiometry values obtained may arise from the concerted binding of several TFP molecules due to the propensity of condensed aromatic ring compounds like TFP and W7 to associate. In line with this, we have previously shown that CaM could accommodate a bundle of lipid molecules in its collapsed conformation.^41^ Alternatively, the allocation of the TFP‐binding sites in the CaM‐4TFP complex allows binding of two additional TFP molecules assuming the structural symmetry of the two protein lobes as already suggested.^39^

No crystal structure with the antagonist W7 has been available yet, however, based on solution NMR data, a 1:2 stoichiometry is widely accepted corresponding to the binding of one small molecule to each hydrophobic pocket.^42^ Fitting our data with the *one set of sites* model assuming several independent, identical binding sites, a stoichiometry *n* of up to 8 could be estimated. However, we also obtained a good fit with fixed *n* = 4 (Figure 7B). The affinity of the CaM‐W7 interaction is reported to be weaker (with *K*_d_ = 11 μmol/L^43^) than that of the CaM‐TFP binding, which is also supported by our data determined here. Specifically, we obtained *K*_d_ values of 7 and 10 μmol/L to W7 for human and Pf CaM, respectively, indicating comparable binding strengths. Nevertheless, the similar binding events with the human and Pf CaM are evident from the almost identical ITC curves for both small molecule interactors (Figure 7A,B).

#### 3.3.2 Melittin binding

Melittin, a 26‐mer basic amphipathic peptide is generally used to model CaM‐target peptide binding. The CaM‐melittin interaction can easily be detected using native PAGE since binding of the peptide bearing six positive charges leads to a remarkably slower migration of the Ca^2+^‐saturated protein, which was shown for both CaMs (Figure 3A). Affinity values in the low nanomolar range (3‐110 nmol/L) were reported for the 1:1 stoichiometry interaction with mammalian CaMs, for example, in Ref. [9, 44, 45] We investigated the binding parameters by means of ITC. Melittin in the cell was titrated with CaM to overcome peptide dilution heat as melittin is known to form tetramers at higher concentrations (in the low and moderate micromolar range, depending on the ionic strength of the medium^46^). The binding curves exhibited steep transitions (Figure 7C) indicative of strong binding so that we could estimate an upper limit for a *K*_d_ of ~15‐30 nmol/L for peptide interactions with both human and Pf CaCaM using the *one set of sites* model. However, the nonconstant initial regime of the calorimetric traces at low CaM‐to‐peptide ratios indicated a more complex binding event for human CaM, which could better fit with the *two sets of sites* model assuming more than one single binding site/mode (Figure 7C). Melittin binding to mammalian CaM has already been examined by ITC, employing very a similar experimental setup as used here, however, no such an observation was reported.^9^

In contrast to small molecule binding characterized by negative enthalpies, positive binding enthalpy values were obtained for the peptide binding suggesting different driving forces for the interactions. Our results with the human protein are in good agreement with a previous work^9^ reporting strikingly different energetics for target peptide binding of CaM, and dominant entropic contribution and hydrophobic interaction for the complex formation with melittin. Moreover, the somewhat reduced Δ*H* values for the interaction with Pf CaM compared with human CaM (Figure 7C) indicated variation in the binding characteristics, presumably due to the different residues forming the hydrophobic pockets.

Nevertheless, the structural determinants of the CaM‐peptide interaction could directly be studied in our crystal structures of melittin complexed with human and Pf CaM,^47^ which will be published in a separate paper.

#### 3.3.3 ANS binding

Substitutions like Met71 in human CaM to Leu in Pf CaM involve residues forming the hydrophobic pockets of CaM. These changes might influence ligand binding properties of the two lobes of CaM as indicated by our melittin binding experiments using ITC. Differences in the hydrophobic nature of the Ca^2+^‐saturated proteins were monitored by ANS binding, which is generally used to probe hydrophobic patches of proteins by detecting changes in its fluorescence.^48^ Human CaCaM showed remarkable ANS binding while Pf CaCaM exhibited even higher, approximately twofold higher fluorescence intensity (Figure 7D). This might be attributed mainly to the Met to Leu substitution in the N‐domain hydrophobic pocket. Other changes (like Met vs Leu and Thr vs Ile at residues 77 and 147, respectively), resulting in elevated overall hydrophobicity of Pf CaM over human CaM might also contribute to increase ANS binding. However, the fact that the apoproteins with closed hydrophobic pockets showed no ANS binding argues against this idea. In contrast, present results indicated that the primer site of the interaction with ANS is represented by the hydrophobic pockets as suggested previously.^5^

#### 3.3.4 CaM activation of target enzymes

CaM regulates the function of over 300 intracellular proteins,^49^ and activates many enzymes. Among them, the phosphatase CaN, the PDE, and the kinase MLCK were tested for in vitro activation by human and Pf CaM.

**FIGURE 8:**
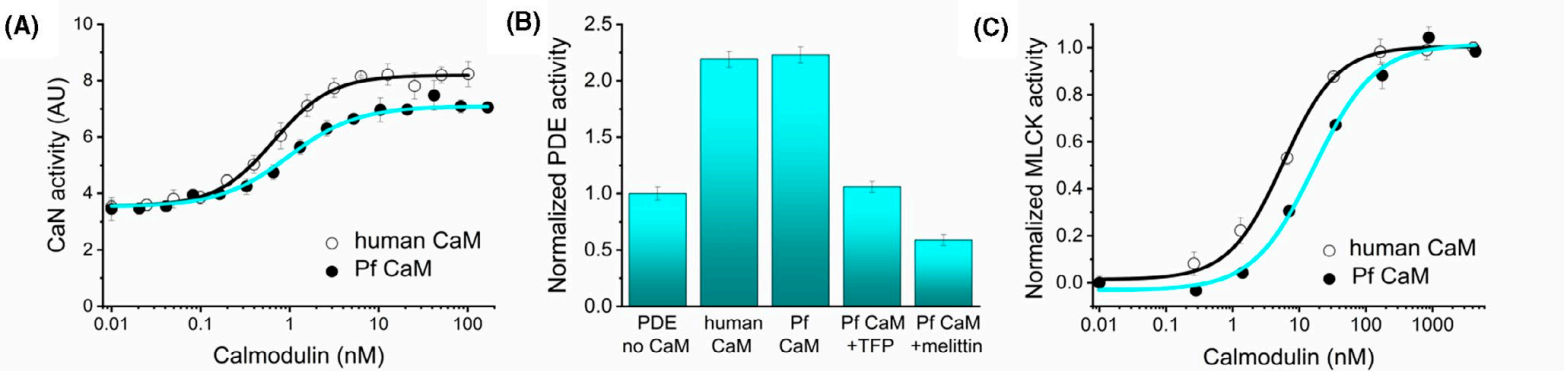
Enzyme activation by human and Pf CaM. Activation ability was tested with CaN (A), PDE (B), and MLCK (C). Activity is shown in arbitrary units (A), normalized to the values measured in the absence of CaM (B), or normalized between zero (for the inactive enzyme) and maximal values in the presence of saturating amounts of CaM (C), respectively. In the case of Pf CaM, PDE activity was also measured in the presence of TFP or melittin (panel B, last two columns). Parameters obtained from fitting to sigmoidal dose‐response curves are A) CaN vs human CaM: EC_50_ = 0.67 ± 0.04 nmol/L, activation by CaM: 2.34‐fold, and CaN vs Pf CaM: EC_50_ = 0.97 ± 0.10 nmol/L, activation by CaM: 2.07‐fold; C) MLCK vs human CaM: EC_50_ = 5.6 ± 0.52 nmol/L, and MLCK vs Pf CaM: EC_50_ = 16.7 ± 2.8 nmol/L

In the case of bovine brain CaN, human CaM induced 2.3‐fold elevation of the basal activity (Figure 8A). Measuring the CaM dependence of the activity, an EC_50_ value of 0.6 nmol/L could be estimated, which is in good agreement with EC_50_ values reported for CaN activation by bovine brain CaM.^50^ Comparing CaM function, Pf CaM increased the CaN activity to a slightly lower extent of 2.1, with a similar EC_50_ value of 1.0 nmol/L (Figure 8A), indicating similar enzyme activation ability for the two CaMs.

Probing bovine PDE, similar activation profiles were measured for human and Pf CaM again (Figure 8B). The enzyme activity increased about twofold in the presence of human CaM, and the same activation effect could be reached upon addition of Pf CaM. In the presence of the antagonist TFP, the basal activity of CaN was measured proving that the activation by CaM was inhibited. Interestingly, an even higher reduction of the activity below the basal level could be measured upon addition of melittin, the reason for that might be the inhibition of PDE by melittin as well. The latter idea is strengthened by the fact that inhibition of enzymatic activity by melittin has already been observed.^51^

CaM binding to the inactive MLCK enzyme induces the kinase activity.^52^ Testing human MLCK, we have determined an EC_50_ value of ~5 nmol/L for the enzyme activation by human CaM, and an approximately threefold higher EC_50_ of ~15 nmol/L was evaluated for Pf CaM indicating slightly reduced activation ability for the Pf protein (Figure 8C).

To sum up the results obtained with the Ca^2+^/CaM‐dependent enzymes tested, we could detect activity induction of the mammalian target enzymes to a similar extent by human and Pf CaM, with slightly reduced capability of Pf CaM.

### 3.4 Antimalarial compounds as CaM antagonists

**FIGURE 9:**
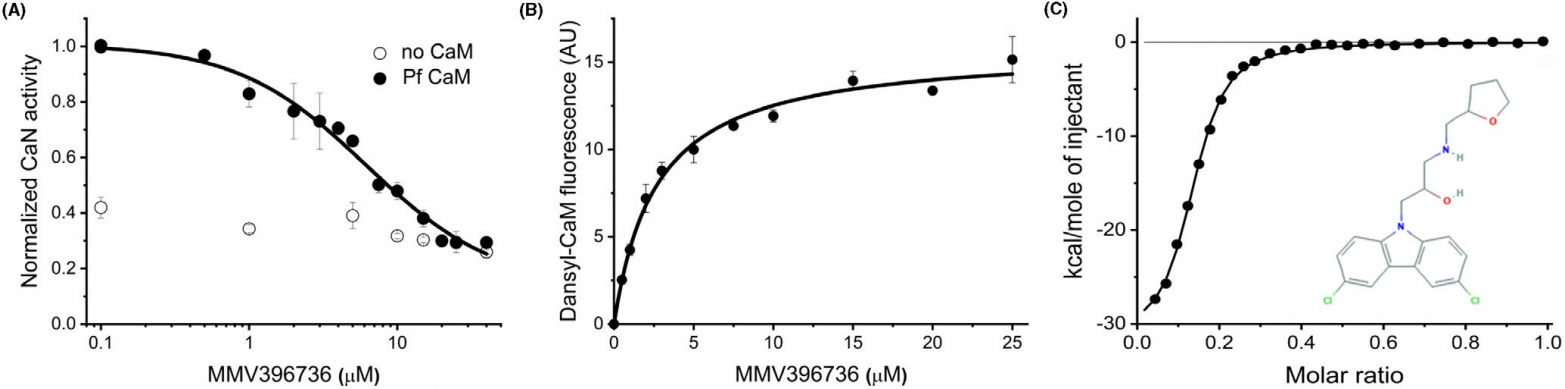
Pf CaM assays with MMV396736. A, The effect of MMV396736 on target enzyme activation was tested on CaN, and an IC_50_ = 6.2 ± 1.5 μmol/L was calculated from fitting to dose‐response function. B, MMV396736 binding to dansyl‐labeled Pf CaM was followed using dansyl fluorescence. Data were fitted to a hyperbolic function, obtaining *K*_d_ = 2.4 ± 0.4 μmol/L. C, MMV396736 was titrated with Pf CaM using ITC. Data were fitted with the *one set of sites* model, yielding n = 7.4 ± 0.1, and *K*_d_ = 2.5 ± 0.2 μmol/L. The chemical structure of the compound MMV396736 is also shown. Image was taken from PubChem (https://pubchem.ncbi.nlm.nih.gov). It is positioned in well G8, plate C (drug‐like molecules) of the Malaria Box (https://www.mmv.org/sites/default/files/uploads/docs/malariabox/PlateC.html)

The Malaria Box is a collection of compounds with proven antimalarial activity, however, mostly with unknown mechanisms of action. Based on the fact that CaM‐antagonists were already reported to exert antimalarial effects, we hypothesized that some compounds in the Malaria Box might have the potential to bind to CaM, and thereby exert their antimalarial activity. Computational analysis using bovine CaM structure as a model predicted an ability of CaM to bind to ~40 small molecules out of the 400 included in the Malaria Box (J. Mestres, personal communication). The compounds selected were tested in vitro for inhibition of Pf CaM function using the CaN activity assay. Four compounds, namely MMV396736, MMV396672, MMV665953, and MMV665902 (for their chemical structures see Figure 9C and Figure S1), showed inhibitory effect, thus were probed for interaction with human and Pf CaM in in vitro binding and functional assays in detail, using compounds purchased from commercial vendors. Finally, the compound MMV396736 only was proven a potent CaM‐antagonist with IC_50_ = 6.2 ± 1.5 μmol/L on CaN (Figure 9A). The dissociation constant of the CaM‐small molecule interaction was characterized by ITC and dansyl‐CaM fluorescence yielding *K*_d_ values in the low micromolar range (Figure 9B,C). Based on the obtained stoichiometry of *n* ~ 8, this compound shares the propensity of multiple binding, also observed for TFP and W7 (see above).

For purposes of comparison, binding experiments were performed with human CaM as well, resulting in comparable affinity values, with slightly stronger binding for the human protein (*K*_d_ of 2.4 ± 0.4 μmol/L vs 1.8 ± 0.4 μmol/L derived from fluorescence‐based titrations using dansyl‐CaM, and 2.5 ± 0.2 μmol/L vs 2.5 ± 0.5 μmol/L obtained from ITC, for Pf vs human CaM, respectively). However, more significant difference was found in CaN assays, where the MMV compound showed approximately fourfold stronger effect on human CaM function (IC_50_ = 6.2 ± 1.5 μmol/L with Pf CaM vs IC_50_ = 1.6 ± 0.76 μmol/L with human CaM).

## 4 DISCUSSION

The calcium‐binding protein CaM is well‐known for its essential roles in Ca‐signaling in higher eukaryotes. The protein is also present in protozoan parasites like the *Plasmodium* species, the causative agents of malaria. It has been observed that CaM antagonists are toxic to malaria parasites. Additionally, CaM was suggested a potential target of some antimalarial compounds of the Malaria Box with mostly unknown mechanism of action. We tested selected compounds for direct binding to CaM from Pf, and compared the affinity of the most promising hit, MMV396736, to that for human CaM. Furthermore, we performed a comparative study of human and Pf CaM involving structural and functional aspects in order to characterize the biophysical and biochemical properties of the proteins to aid potential drug design.

CaM is a highly conserved protein, which is clearly illustrated by the fact the human and Pf homologs share 89% sequence identity despite their distant relationship. Many of the 16 substitutions (out of the total 148 residues, Figure 1) are localized in the more flexible protein parts: the central region involving the flexible linker connecting the two lobes, and within the terminal parts. Nevertheless, several changes can be found in the EF‐hands coordinating calcium ions, suggesting alterations in affinity to Ca^2+^ and related protein folded state. Indeed, slight differences were revealed for the two proteins in both the basic conformation of the apo‐ and CaCaM and the nature of the conformation change upon Ca^2+^ binding (Figures 2, 3, 4).

Most of the differing residues are similar substitutions, and some changes involve methionines in the human sequence (Figure 1). Mammalian CaM with its nine Met residues (6.1%) is especially rich in methionines with key roles in both the structural flexibility and the formation of the hydrophobic pockets responsible for ligand binding plasticity. This is clearly verified in human CaM variants with all the four Mets in the C‐domain engineered to Leu, where the substitutions resulted in enhanced protein stability indicated by dramatically increased *T*_m_ values.^5^ Here we observed the same phenomenon for Pf CaM compared to human CaM (Figures 5, 6, Table 1), although there are only two Met‐to‐Leu changes in Pf CaM, Met71 in the N‐domain and Met76 in the central linker region, suggesting primary roles for these Mets in structural stability.

In vertebrate CaMs, each hydrophobic pocket is formed by four hydrophobic residues, namely a Phe, a Leu, and two Mets. The pockets can be considered as primary binding sites for the hydrophobic probe ANS (Ref. [53] and the present study), since no change in ANS fluorescence was detected for the apo form where pockets are closed (Figure 7D). Probing ANS to CaCaM, we have observed more remarkable ANS binding for Pf CaCaM over human CaCaM as indicated by elevated fluorescence intensities and larger blue‐shift for Pf CaCaM‐bound ANS (Figure 7D). Similar effect was found for Met‐to‐Leu substituted human CaM.^5^ For the latter case, ANS binding was also examined by ITC obtaining slightly increased affinity as well as higher exothermic interaction heats, which was attributed to deeper binding of ANS into the C‐domain hydrophobic pocket due to the shorter Leu side chains forming the clefts, compared to that of Mets. This consideration might hold for our case as well, although only one Met in the N‐domain pocket has changed to Leu in Pf CaM. This change in the hydrophobic pockets of Ca^2+^‐loaded human and Pf CaMs might result in altered ligand‐binding properties due to the importance of hydrophobic contacts in accommodating its partner molecules by CaM.

Further on, we have investigated the ligand‐binding properties of human and Pf CaM using small molecule antagonists, which can be considered as model drugs to block CaM action. The almost identical binding isotherms obtained for the interaction with W7 and TFP (Figure 7A,B) argue for similar binding events with the two proteins. Moreover, the Malaria Box antimalaria compound MMV396736 identified here as novel potent CaM‐antagonist also shared the similarity in its interaction with the two proteins of different origin (Figure 9 and related text).

For each Malaria Box compound, toxicity results obtained from in vitro screening against 3D7 chloroquine sensitive but sulfadoxine resistant strain of *P falciparum* in the blood stage are available at the MMV website (https://www.mmv.org/mmv‐open/malaria‐box/malaria‐box‐supporting‐information). Accordingly, MMV396736 exhibited an EC_50_ of 1.06 μmol/L on *P falciparum*. Compared to the affinity determined here for its CaM binding (*K*_d_ of 2.5 μmol/L in direct binding assays, Figure 9), inhibition of CaM by MMV396736 could contribute to the toxicity observed on the parasite. Considering drug design, based on its physicochemical properties, MMV396736 was selected to the *drug‐like* set of Malaria Box. These could make MMV396736 a promising drug hit against malaria.

In contrast to highly similar small molecule binding detailed above, more remarkable variations in the binding determinants could be deduced for the CaM‐target peptide interaction based on the example with the model peptide melittin. Overall, results indicated similar apparent binding affinities assuming the 1:1 complex with each CaM. However, ITC data at low CaM‐to‐peptide ratios (Figure 7C) suggested a more complex binding event with human CaM, with two potential peptide binding sites––or modes––on the protein. Further on, the unfavorable enthalpy contribution of the binding was reduced in the case of Pf CaM (Figure 7C), which might be attributed to changes in the hydrophobic pockets. A detailed structural insight on characteristics of target peptide binding will be provided based on the crystal structures of their melittin complexes in a separate publication.

To explore activation capacity, we have performed in vitro functional assays with human and Pf CaM using Ca^2+^/CaM‐dependent enzymes. Probing bovine CaN, bovine PDE, and human MLCK, similar ability for enzyme activation was obtained for the human and parasite‐derived CaMs, with slightly weaker capability of Pf CaM. (Figure 8). However, more pronounced difference in antagonistic effect was detected when probing the potent Malaria Box compound, MMV396736, on CaM function as it showed fourfold higher inhibition potency with the human protein in CaN assay (Figure 9 and related text). Nevertheless, the biological origin of the CaM‐dependent enzymes might affect the relative activation ability observed here. As bovine and human CaMs are identical, and the bovine and human CaN and PDE homologs differ only in a few residues, these slight variations are not expected to remarkably affect the response of the CaMs investigated. Comparing the human and *Plasmodium* homologs of the target enzymes, *Plasmodium* species express a CaN‐like enzyme, and also cyclic nucleotide‐specific PDEs, however, implicitly, no MLCK. A functional study with the *Plasmodium* enzymes could presumably reveal a preference to the parasite CaM over the vertebrate variant. However, the functional similarity of the CaMs of different origin suggested also by our findings argues for only small differences to be expected.

Taken together, our data demonstrated that changes in the primary sequence had only small effect on the target‐/ligand‐binding properties of human and Pf CaM. This finding is consistent with the function of CaM exploiting plasticity to bind to multiple targets, and also in agreement with results using various CaM mutants where substitutions resulted in no significant alterations in affinity to various partners, compared with the wild‐type protein.^54^, ^55^ As typically higher difference in affinity is needed to reach the required selectivity toward the pathogen to combat in comparison to effects exerted on the host organism, unfortunately, our results point to potential serious toxic side effects when using compounds targeting a key modulator protein like CaM. Our experiments confirm the modeling‐based suggestion of CaM being a non‐suitable target for novel antimalarials.^56^

In contrast to the slightly modified ligand‐binding properties detailed above, mutations can lead to dramatic changes in protein stability. Indeed, we could detect markedly enhanced stability for both the apo and Ca^2+^‐loaded forms of Pf CaM over the human variant using various methods, such as DSC, CD, and DSF (Figures 5, 6 and Table 1). The increased stability is probably linked to substitutions resulting in higher hydrophobic content, which is supported by several data as follows. A shift in the SDS‐PAGE migration pattern, similarly to that shown here for Pf CaM compared to human CaM (Figure 4), was reported for CaM from another protozoan, *T pyriformis*, displaying substitutions for example, Met71‐to‐Leu, Arg86‐to‐Ile, Thr146‐to‐Leu, or to Met.^30^ Moreover, vertebrate CaMs with methionines replaced to leucines were shown to be stabilized against heat denaturation^5^ as observed here for Pf CaM in comparison to human CaM. A computational study on CaM stability revealed the importance of one pair of hydrophobic residues in each lobe appearing critical in force‐induced unfolding, and suggested their involvement in thermal denaturation as well.^3^ Though the Ile27‐Ile63 pair in the N‐lobe is present in both the human and Pf proteins, the Ile100‐Val136 pair in the C‐lobe of the human sequence is changed to Ile100‐Ile136 in Pf CaM. The Ile‐Ile interaction could be beneficial due to the higher total hydrophobic area, thereby contributing to the enhanced stability of Pf CaM.

In consideration of the conformational stability, we can conclude that it is a multivariant issue for the two‐domain protein CaM composed of four calcium‐binding sites with various affinity to Ca^2+^ ions. Stability is dictated by the folded state, related hydrophobic contacts, and calcium affinity. Importance of the folded state is highlighted for the apoprotein in which the more folded N‐domain exhibits the high stability domain. However, enhanced hydrophobic contacts presumably coupled to folding can also contribute significantly as observed for the C‐domain of Pf apoCaM compared to the human protein variant. Calcium binding has an overall dramatic effect on the stability reflected in the remarkably elevated melting points for both lobes. Further on, calcium affinity of the individual EF‐hand motifs seems to play key roles too, as our results point to a higher stability C‐domain in the Ca^2+^‐loaded protein. Nevertheless, hydrophobic content could have an effect again which is supported by the further increased thermal stability of the C‐domain of Pf CaM over human CaM.

## 5 CONCLUSION

The present comparative study on human and *P falciparum* CaM demonstrated fairly similar ligand‐binding properties for the two proteins, despite the high evolutionary distance of their species. In contrast, we also showed that replacement of a few amino acids can lead to remarkably differences in stability, in spite to high similarity in the protein structure. Herein, we also report that Malaria Box compounds can exert potent CaM‐antagonist activity, connecting mechanism of action of antimalarial agents to inhibition of CaM function. Nevertheless, due to the minor alterations found in the affinity of human and Pf proteins toward these compounds, development of a lead against malaria should focus on other targets where the selectivity needed between the host and pathogen could be reached easier.

## AUTHOR CONTRIBUTIONS

T. Juhász and K. Liliom designed research; T. Juhász and J. Kardos performed research; T. Juhász and J. Kardos analyzed data; Z. Dürvanger and V. Harmat prepared figures and contributed to interpretation of results, and all authors contributed to writing the manuscript.

## Supporting information

Fig S1
