# Supplementary material for: Comparison of ligand binding and conformational stability of human calmodulin with its homolog from the malaria parasite Plasmodium falciparum
Source: FASEB Bioadv. 2020 Aug 9;2(8):489–505. doi: 10.1096/fba.2020-00013 (PMC7429351; doi:10.1096/fba.2020-00013)
Supplement: Supplementary file 1 — Fig S1 [file FBA2-2-489-s001.pdf]

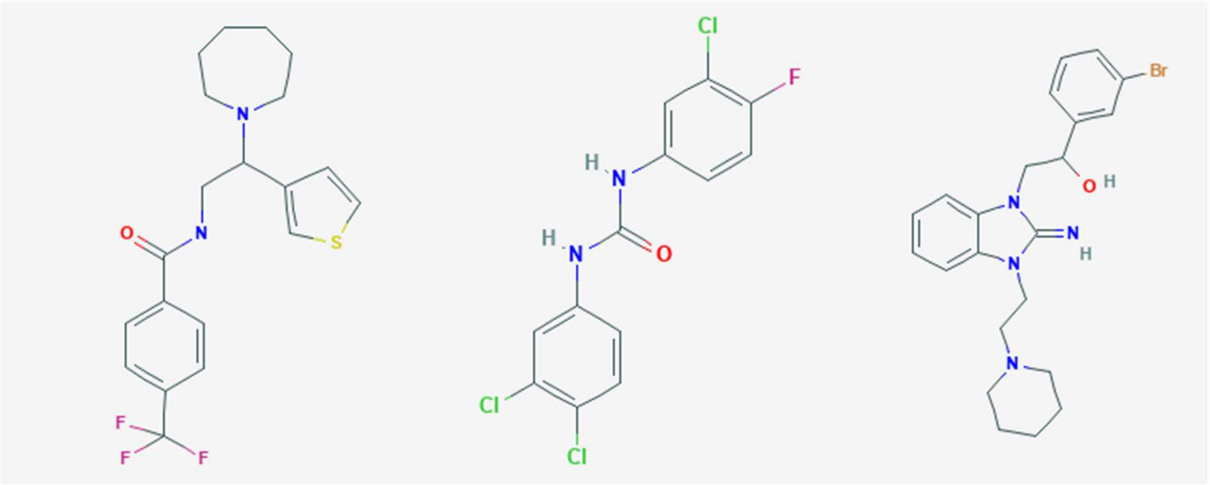

MMV396672 | MMV665953 | MMV665902

Plate A well A10 | Plate B well D4 | Plate B well C8

Figure S1. **Malaria Box compounds showing inhibition on Pf CaM in calcineurin functional assays.** The positions according to the Malaria Box plates are also indicated. Plate A contains the 40 most active drug-like molecules and the 40 most active probe-like molecules whereas Plate B contains 80 drug-like molecules. Structures were taken from PubChem.

https://www.mmv.org/sites/default/files/uploads/docs/malariabox/PlateA.html
https://www.mmv.org/sites/default/files/uploads/docs/malariabox/PlateB.html
